# Social Participation and Dental Health Status among Older Japanese Adults: A Population-Based Cross-Sectional Study

**DOI:** 10.1371/journal.pone.0061741

**Published:** 2013-04-17

**Authors:** Kenji Takeuchi, Jun Aida, Katsunori Kondo, Ken Osaka

**Affiliations:** 1 Department of International and Community Oral Health, Tohoku University Graduate School of Dentistry, Miyagi, Japan; 2 Center for Well-being and Society, Nihon Fukushi University, Aichi, Japan; University of Toronto, Canada

## Abstract

**Background:**

Although social participation is a key determinant of health among older adults, few studies have focused on the association between social participation and dental health. This study examined the associations between social participation and dental health status in community-dwelling older Japanese adults.

**Methods and Findings:**

In 2010, self-administered postal questionnaires were distributed to all people aged ≥65 years in Iwanuma City, Japan (response rate, 59.0%). Data from 3,517 respondents were analyzed. Data on the number of remaining natural teeth, for determining the dental health status, and social participation were obtained using self-administered questionnaires. The number, type, and frequency of social activities were used to assess social participation. Social activities were political organizations or associations, industrial or professional groups, volunteer groups, senior citizens' clubs, religious groups or associations, sports groups, neighborhood community associations, and hobby clubs. Using ordinal logistic regression, we calculated the odds ratios (OR) and 95% confidence intervals (95% CI) for an increase in category of remaining teeth based on the number, type, and frequency of social activities. Sex, age, marital status, current medical history, activity of daily living, educational attainment, and annual equivalent income were used as covariates. Of the respondents, 34.2% reported having ≥20 teeth; 27.1%, 10–19 teeth; 26.3%, 1–9 teeth; and 12.4%, edentulousness. Social participation appeared to be related with an increased likelihood of having a greater number of teeth in old age, even after adjusting for covariates (OR = 1.30, 95% CI = 1.10–1.53). Participation in sports groups, neighborhood community associations, or hobby clubs was significantly associated with having more teeth.

**Conclusions:**

Our results suggest a protective effect of social participation on dental health. In particular, participation in sports groups, neighborhood community associations, or hobby clubs might be a strong predictor for retaining more teeth in later life.

## Introduction

Enhanced social participation, a social determinant of health [1], [2], is one of the 3 pillars of a World Health Organization (WHO) policy framework for an active aging society [3]. Social participation is a source of social relations and describes a person's participation in formal and informal group activities [4], [5], [6]. As many older retired people are assumed to have more time to participate in other activities, the role of social participation in the health of older adults is increasing in today's aging society.

Previous studies have examined the association between social participation and various health outcomes. A meta-analysis determined that social participation reduced the risk for mortality and that the magnitude of this effect was comparable with smoking cessation [7]. A study conducted in Asia reported that maintaining or initiating social participation in later life benefited the mental health of older adults [8]. A study conducted in Japan reported that lack of social participation was significantly related to an increased risk for onset of long-term care insurance certification [9]. In addition to the effect itself, social participation is important because it is a component of social capital [10]. According to Putnam, social capital refers to “features of social organization such as networks, norms, and social trust that facilitate coordination and cooperation for mutual benefit” [11]. Recent studies have demonstrated a positive association between social capital and various health outcomes, including dental health [12], [13], [14], [15], [16], [17].

Social participation is also considered to affect dental health [18], [19]. Previous studies have demonstrated that lower levels of social participation were associated with a higher risk for edentulism [18] or periodontitis [19]. There are 2 plausible relationship mechanisms between social participation and dental health: social network as a main effect, and stress buffering [20]. The main effect of social participation is obtained from social relationships, and this mechanism is beneficial regardless of whether individuals are under stress. Participation in a broad range of social relationships develops a person's social network. Individuals in a social network are subject to social controls and peer pressure that influence normative dental health behaviors (e.g., developing good dental habits and quitting smoking). For example, the cessation of smoking in one person appears to be highly related to the smoking behavior of others nearby in that person's social network [21]. Social network ties also provide multiple sources of information that could influence behaviors relevant to oral health, result in the effective use of available dental health services, or help people avoid stressful or other high-risk situations. In addition to this main effect, stress buffering is also considered a pathway to good dental health. A systematic review of the literature suggests that psychological stress causes periodontal disease, which is a key risk factor for tooth loss [22]. Social networks are a source of social support, which in turn provides psychological and material resources intended to benefit an individual's ability to cope with stress. As social support promotes less threatening interpretations of adverse events and effective coping strategies, it can shield individuals from the effects of stressful experiences. This mechanism is called stress buffering.

Despite a recent increase in studies on social participation and health, only a small number of studies have focused on the association between social participation and oral health. In addition, previous oral epidemiological studies have defined social participation as only belonging or not belonging to social relationships, or as high or low frequency of social engagement. A meta-analysis revealed that definitions of social participation mostly focused on questions of who, how, what, with whom, and where [6]. To our knowledge, the present study is the first to focus on the number, type, and frequency of social activities. This study aimed to quantify the associations between social participation and dental health status in community-dwelling older Japanese adults.

## Methods

### Study sample

The present analysis was based on a subset of the Japan Gerontological Evaluation Study (JAGES) project data. The JAGES project is an ongoing prospective cohort study investigating factors associated with the loss of health related to functional decline or cognitive impairment among individuals aged 65 years or older. In 2010, self-administered postal questionnaires were distributed to all people aged ≥65 years in Iwanuma City, Miyagi Prefecture, Japan (n = 8,576), and 5,058 (response rate, 59.0%) people returned the questionnaires. After excluding respondents who failed to provide information on sex, dental health status, or social participation, the data from 3,517 respondents were analyzed. If the respondents did not respond to the other variables, the corresponding observations were assigned to “missing” categories. Ethical approval for the study was obtained from the Ethics Committee at Tohoku University and Nihon Fukushi University.

### Outcome variable

The number of remaining natural teeth, derived from responses collected through the self-administered questionnaire, was used as an indicator of dental health status. Respondents were asked to classify their dental health status into one of 4 categories: ≥20 teeth remaining, 10–19 teeth remaining, 1–9 teeth remaining, or no teeth remaining.

### Main predictors

Social participation was defined as the person's involvement in social activities. First, respondents were asked whether they belonged to political organizations or associations, industrial or professional groups, volunteer groups, senior citizens' clubs, religious groups or associations, sports groups, neighborhood community associations, or hobby clubs. Second, respondents were asked to indicate the frequency of participation in each group: 2–3 times per week, once per week, several times per month, several times per year, or almost never. As there were very few “2–3 times per week” responses for 6 groups (political organizations or associations, industrial or professional groups, volunteer groups, senior citizens' clubs, religious groups or associations, and neighborhood community associations), we re-categorized these social participation variables: once or more per week, several times per month, several times per year, almost never. As our study also focused on the number of social activities, we calculated the numbers of social activities and created 6 categories: 0 groups, 1 group, 2 groups, 3 groups, 4 groups, and ≥5 groups.

### Covariates

It was assumed that physical health status was associated with both social participation and dental health status. Activity of daily living and current medical history were used as indicators of physical health status. Activity of daily living was categorized as independent, partially dependent, and dependent. Current medical history was measured by the question, “Do you receive treatment now?” to which respondents answered “yes” or “no.” Sex, age, and marital status were used as socio-demographic characteristics. Age groups were categorized as 65–69, 70–74, 75–79, 80–84, and ≥85 years. Marital status was categorized as married, widowed, separated, never married, and other. Educational attainment and annual equivalent income were used as indicators of socioeconomic status. Educational attainment was categorized as <6, 6–9, 10–12, and ≥13 years. Annual equivalent income was divided into quartiles: lowest, low-middle, high-middle, and highest.

### Statistical analysis

Descriptive statistics were used to characterize the respondents. We performed ordinal logistic regressions to examine the associations between social participation and dental health status. We calculated the odds ratios (OR) and 95% confidence intervals (95% CI) for an increase in the remaining teeth category based on the number, type, and frequency of social activities. To estimate the overall effect of social participation, we used a dichotomized variable of social participation (1 = participating in ≥1 groups, 0 = not participating in any group). Variables on social participation were included separately in the different models. In the univariate model (Model 1), we calculated the crude OR for dental health status based on the number of social activities and the type and frequency of social participation. In the multivariable model (Model 2), we added all covariates into the univariate model. In order to assess the public health impact of social participation, we calculated the population-attributable fraction (PAF) of having ≥20 teeth to social participation. The PAF is generally defined as the reduction in the burden of disease (or risk factor) that would be achieved if the population had been entirely unexposed, compared with its current exposure pattern [23]. In this study, we treated the PAF as the increase in the number of people with ≥20 remaining teeth that would be achieved if the entire population participated in some kind of social group, compared with its current participation pattern. We calculated a PAF for ≥20 remaining teeth because the retention of a minimum of 20 functional natural teeth at the age of ≥65 years is a goal for oral health specified by the WHO/*Federation Dentaire Internationale* in 2000 [24]. The goal for an acceptable level of oral health determined by the Japan Dental Association is the retention of at least 20 functional teeth until the age of 80 years (8020 movement). A previous study also indicated that among older people, those with ≥20 teeth had higher food intakes than those with ≤19 teeth [25]. All analyses were performed using SPSS statistical software (version 17.0, SPSS, Chicago, IL).

## Results

The demographic and health characteristics of all respondents (n = 3,517; average age, 73.5 years for men and 75.0 years for women) in the study are shown in Tables 1 and 2. Of the respondents, 34.2% reported having ≥20 teeth, 27.1% reported having 10–19 teeth, 26.3% reported having 1–9 teeth, and 12.4% reported having no teeth. Of the respondents, 13.9% belonged to political organizations or associations, 15.2% to industrial or professional groups, 16.4% to volunteer groups, 15.7% to senior citizens' clubs, 7.3% to religious groups or associations, 24.5% to sports groups, 46.8% to neighborhood community associations, and 41.1% to hobby clubs.

**Table 1:** Characteristics of respondents.

	n	%		n	%
**Sex**			**Educational attainment (years)**		
Men	1,681	47.8	<6	86	2.4
Women	1,836	52.2	6–9	1,071	30.5
**Age (years)**			10–12	1,521	43.2
65–69	1,147	32.6	≥13	762	21.7
70–74	950	27.0	Missing	77	2.2
75–79	649	18.5	**Annual equivalent income (quartiles)**		
80–84	418	11.9	Lowest	718	20.4
≥85	346	9.8	Low-middle	731	20.8
Missing	7	0.2	High-middle	801	22.8
**Marital status**			Highest	792	22.5
Married	2,416	68.7	Missing	475	13.5
Widowed	855	24.3	**Number of remaining natural teeth**		
Separated	111	3.2	≥20	1,203	34.2
Never married	50	1.4	10–19	952	27.1
Other	28	0.8	1–9	925	26.3
Missing	57	1.6	No	437	12.4
**Current medical history**			**Number of social activities (groups)**		
Yes	2,741	77.9	0	1,068	30.4
No	731	20.8	1	749	21.3
Missing	45	1.3	2	644	18.3
**Activity of daily living**			3	456	13.0
Independent	3,155	89.7	4	281	8.0
Partially dependent	208	5.9	≥5	319	9.1
Dependent	122	3.5			
Missing	32	0.9			

**Table 2:** Characteristics of respondents according to type and frequency of social participation.

	2–3 times per week n (%)	Once per week n (%)	Several times per month n (%)	Several times per year n (%)	Almost never n (%)
**Type and frequency of social participation**					
Political organization or association	45 (1.3)	32 (0.9)	90 (2.6)	321 (9.1)	3,029 (86.1)
Industrial or professional group	56 (1.6)	36 (1.0)	126 (3.6)	318 (9.0)	2,981 (84.8)
Volunteer group	52 (1.5)	59 (1.7)	192 (5.5)	275 (7.8)	2,939 (83.6)
Senior citizens' club	27 (0.8)	61 (1.7)	185 (5.3)	280 (8.0)	2,964 (84.3)
Religious group or association	23 (0.7)	34 (1.0)	81 (2.3)	120 (3.4)	3,259 (92.7)
Sports group	259 (7.4)	245 (7.0)	183 (5.2)	176 (5.0)	2,654 (75.5)
Neighborhood community association	44 (1.3)	61 (1.7)	282 (8.0)	1,260 (35.8)	1,870 (53.2)
Hobby club	284 (8.1)	350 (10.0)	500 (14.2)	311 (8.8)	2,072 (58.9)

Of all respondents, 69.6% participated in ≥1 groups, and 30.4% did not participate in any group. Compared to the non-participants, participants had significantly higher odds of having a greater number of teeth (OR = 2.40, 95% CI = 2.10–2.74). After adjusting for sex, age, marital status, current medical history, activity of daily living, educational attainment, and annual equivalent income, social participation appeared to be related with an increased likelihood of having a greater number of teeth in old age (OR = 1.30, 95% CI = 1.10–1.53).

Table 3 illustrates the association between dental health status and the number of social activities. Participating in ≥1 groups was significantly associated with odds of having more remaining teeth that were more than twice as high as compared with non-participation (Model 1). After adjusting for all covariates, participating in 4 groups was associated with significantly higher odds (OR = 1.46, 95% CI = 1.11–1.93) of having more remaining teeth compared with non-participation (Model 2). Table 4 shows the association between dental health status and the type and frequency of social participation. The groups significantly associated with a higher number of remaining teeth were industrial or professional groups, volunteer groups, sports groups, neighborhood community associations, and hobby clubs (Model 1). After adjusting for all covariates, participating in sports groups (2–3 times per week, OR = 1.31, 95% CI = 1.01–1.69), neighborhood community associations (several times per year, OR = 1.19, 95% CI = 1.02–1.39), or hobby clubs (2–3 times per week, OR = 1.36, 95% CI = 1.05–1.76; once per week, OR = 1.39, 95% CI = 1.10–1.75; several times per year, OR = 1.41, 95% CI = 1.11–1.81) was significantly associated with having more teeth (Model 2). With the exception of these 3 groups, although most types of participation were associated with higher odds of having more teeth, the associations were explained by covariates. This indicates that healthier people tend to have more teeth and participate in groups.

**Table 3:** Association of dental health status with number of social activities determined by ordinal logistic regression.

	Model 1	Model 2		
	Crude OR (95% CI)	Adjusted OR a (95% CI)	n of ≥20 teeth (%)	PAF^b^ (%)
**Number of social activities (groups)**				31.6
0	1.00	1.00	250 (23.4)	
1	2.21 (1.86–2.62)	1.31 (1.07–1.59)	279 (37.2)	
2	2.22 (1.85–2.65)	1.21 (0.98–1.49)	231 (35.9)	
3	2.84 (2.32–3.48)	1.36 (1.07–1.72)	194 (42.5)	
4	2.90 (2.28–3.70)	1.46 (1.11–1.93)	125 (44.5)	
≥5	2.31 (1.84–2.90)	1.25 (0.96–1.62)	124 (38.9)	

OR = odds ratio; CI = confidence interval.

a Odds ratio adjusted for sex, age, marital status, current medical history, activity of daily living, educational attainment, and annual equivalent income.

b Population-attributable fraction.

**Table 4:** Association of dental health status with type and frequency of social participation determined by ordinal logistic regression.

	Model 1	Model 2		
	Crude OR (95% CI)	Adjusted OR^a^ (95% CI)	n of ≥20 teeth (%)	PAF^b^ (%)
**Type and frequency of social participation**				
*Political organization or association*				1.4
Once or more per week	1.15 (0.77–1.74)	0.97 (0.61–1.53)	26 (33.8)	
Several times per month	1.33 (0.91–1.95)	1.06 (0.69–1.61)	35 (38.9)	
Several times per year	1.14 (0.93–1.41)	0.89 (0.70–1.11)	120 (37.4)	
Almost never	1.00	1.00	1,022 (33.7)	
*Industrial or professional group*				3.6
Once or more per week	1.29 (0.88–1.87)	1.03 (0.68–1.58)	33 (35.9)	
Several times per month	1.75 (1.26–2.44)	1.17 (0.82–1.67)	55 (43.7)	
Several times per year	1.51 (1.22–1.87)	1.05 (0.83–1.32)	132 (41.5)	
Almost never	1.00	1.00	983 (33.0)	
*Volunteer group*				4.3
Once or more per week	1.38 (0.98–1.96)	1.11 (0.76–1.61)	44 (39.6)	
Several times per month	1.85 (1.41–2.42)	1.31 (0.97–1.76)	89 (46.4)	
Several times per year	1.37 (1.10–1.72)	1.02 (0.79–1.31)	108 (39.3)	
Almost never	1.00	1.00	962 (32.7)	
*Senior citizens' club*				−1.7
Once or more per week	0.76 (0.52–1.12)	0.89 (0.58–1.36)	27 (30.7)	
Several times per month	0.77 (0.59–1.01)	0.76 (0.56–1.02)	58 (31.4)	
Several times per year	0.80 (0.65–1.00)	0.89 (0.70–1.14)	87 (31.1)	
Almost never	1.00	1.00	1,031 (34.8)	
*Religious group or association*				0.4
Once or more per week	0.99 (0.61–1.58)	0.87 (0.51–1.48)	18 (31.6)	
Several times per month	1.06 (0.71–1.58)	1.07 (0.68–1.68)	28 (34.6)	
Several times per year	1.34 (0.96–1.87)	1.31 (0.90–1.90)	47 (39.2)	
Almost never	1.00	1.00	1,110 (34.1)	
*Sports group*				7.5
2–3 times per week	1.90 (1.50–2.41)	1.31 (1.01–1.69)	115 (44.4)	
Once per week	1.73 (1.36–2.20)	1.20 (0.92–1.56)	104 (42.4)	
Several times per month	1.64 (1.25–2.16)	0.99 (0.74–1.34)	75 (41.0)	
Several times per year	1.54 (1.17–2.04)	1.02 (0.75–1.39)	69 (39.2)	
Almost never	1.00	1.00	840 (31.7)	
*Neighborhood community association*				14.5
Once or more per week	1.42 (0.99–2.02)	0.98 (0.65–1.47)	34 (32.4)	
Several times per month	1.63 (1.30–2.05)	0.93 (0.72–1.19)	100 (35.5)	
Several times per year	1.83 (1.60–2.08)	1.19 (1.02–1.39)	522 (41.4)	
Almost never	1.00	1.00	547 (29.3)	
*Hobby club*				16.8
2–3 times per week	1.98 (1.58–2.49)	1.36 (1.05–1.76)	122 (43.0)	
Once per week	2.06 (1.67–2.54)	1.39 (1.10–1.75)	157 (44.9)	
Several times per month	1.84 (1.54–2.20)	1.16 (0.95–1.42)	194 (38.8)	
Several times per year	2.13 (1.71–2.65)	1.41 (1.11–1.81)	140 (45.0)	
Almost never	1.00	1.00	590 (28.5)	

OR = odds ratio; CI = confidence interval.

a Odds ratio adjusted for sex, age, marital status, current medical history, activity of daily living, educational attainment, and annual equivalent income.

b Population-attributable fraction.

The PAFs, or the contribution of social participation to having ≥20 teeth, are shown in Tables 3 and 4. The PAFs for the number of social activities and 3 types of social participation variables that were significantly associated with dental health (i.e., sports groups, neighborhood community associations, and hobby clubs) were 7.5%–31.6%. The largest PAF (31.6%) was for participation in ≥1 social groups.

## Discussion

Our study demonstrates a significant positive association between social participation and dental health status in a representative sample of men and women aged ≥65 years in a municipality in Japan. Among those with ≥20 remaining teeth, 31.6% of cases in the population might be attributed to participation in ≥1 social groups. To our knowledge, no published reports have examined the associations between dental health status and the number, type, and frequency of social activities. In relation to the type and frequency of social participation, frequent participation in sports groups, rare participation in neighborhood community associations, or participation in hobby clubs with little regard to frequency were significantly associated with dental health status, even after adjusting for demographic variables and social class indicators. In relation to the number of social activities, almost all amounts of social participation were significantly positively associated with dental health.

Our results may support the earlier-described mechanisms linking social participation and dental health status (i.e., social network as a main effect and stress buffering). There was a significant association with better dental health status for participants in the groups with higher social participation rates. In groups with high participation rates that include many social ties, people may easily develop social networks and receive social support.

In addition to these positive effects of social participation on health, social participation can have negative effects on health. Social networks provide opportunities for conflict, exploitation, stress transmission, misguided attempts to help, and feelings of loss and loneliness [20]. These potentially negative aspects of social networks can cause psychological stress, which in turn adversely affects dental health. The results of this study showed no significant association between frequent participation in neighborhood community associations and dental health, but there was a significant association between relatively rare participation and dental health. The negative effects of social participation on health may be a reason for this. Participation in neighborhood community associations might include obligatory activities characterized by the negative aspects of social networks. People who frequently participate in obligatory activities may experience stress, leading to oral disease. Therefore, frequent participation in neighborhood community associations might not be significantly associated with having more teeth. Similarly, where participation in ≥5 groups is concerned, social participation might not be significantly associated with having more teeth for an increase in the type of social participation that has negative effects on health.

Our findings are generally consistent with those of previous studies indicating that participating in social activities benefits dental health status among middle-aged and older people. Rodrigues et al. suggested that social participation is significantly associated with a lower prevalence of edentulism among older adults [18]. Merchant et al. also suggested that men who participate in religious meetings are associated with a reduced risk of developing periodontitis [19].

To our knowledge, no study has specifically examined the differences between men and women in relation to the association between social participation and dental health status, though previous work has indicated that such differences exist. Among women, participation in social networks may increase levels of psychological stress [26]. In our study, 75.3% of men participated in ≥1 groups compared to 64.5% of women. However, with respect to the main results, we found few differences between men and women.

The results of this study have public health implications. Our goal was to estimate the PAF associated with participation in social activities (compared to non-participation) for having ≥20 remaining teeth. The largest PAF (31.6%) was for participation in ≥1 social groups, which implies that in 31.6% of cases in the population, presence of ≥20 remaining teeth may be attributed to participation in ≥1 social groups. Similarly, the PAFs for participation in sports groups, neighborhood community associations, and hobby groups were 7.5%, 14.5%, and 16.8%, respectively, for having ≥20 remaining teeth. Therefore, promoting and supporting opportunities for social participation, especially in sports groups, neighborhood community associations, or hobby clubs, as a public health intervention may contribute to an increase in the number of older people with ≥20 remaining teeth.

Our study has several limitations as well as strengths. First, the response rate was moderate (59.0%); hence, our results may have been affected by selection bias. Second, our research data were derived from self-reported questionnaires, raising issues of information bias regarding the true number of remaining teeth. However, self-reports have yielded reasonably valid estimates for the number of teeth in national epidemiological surveys in several prior studies [27], [28]. In a study of 2,496 Japanese older people, the difference between the self-reported number of teeth and the clinically examined number of teeth was very small and insignificant according to the *t*-test, and the correlation between the 2 groups was very high (r = 0.93) [28]. Therefore, it is reasonable to assume that self-reported questionnaires can provide sufficiently reliable data about the number of remaining teeth. Third, our study was cross-sectional; therefore, it was not possible to generate any statements on causation. The present cross-sectional study could not exclude the possibility of reverse causation, in that people with good oral health tend to participate in social activities. Longitudinal studies or intervention studies are needed to examine the effects of social participation on dental health status. Lastly, our study participants were from one medium-sized municipality in Japan; hence, the generalizability of our results is limited. Caution should be exercised when interpreting our results, as it requires the somewhat strong assumption that the data we used for our analysis are generalizable to the entire population.

However, the population sample also could be considered a strength, as our data were less affected by geographic and cultural factors between municipalities, which provides some assurance of internal validity. The level of detail included in the social participation variable is another strength. Lastly, our data were reliable because all of the older residents in one city were recruited for our survey, and a large number of people participated.

## Conclusion

Social participation was significantly and positively associated with better dental health status among older Japanese adults. Approximately one-third of the participants had ≥20 teeth, which may have been attributable to their participation in ≥1 social groups, though the present cross-sectional design could not exclude the possibility that people with good oral health tend to participate in social activities. In addition, our results indicate the possibility that participation in sports groups, neighborhood community associations, or hobby clubs in later life is protective of dental health beyond individual differences in demographic variables and social class indicators.
